# *In vitro* and *in vivo* evaluations of *Pelargonium roseum* essential oil activity against *Trichomonas gallinae*

**Published:** 2018

**Authors:** Mohaddeseh Abouhosseini Tabari, Mohammad Reza Youssefi

**Affiliations:** 1 * Faculty of Veterinary Medicine, Amol University of Special Modern Technologies, Amol, Iran*; 2 *Department of Veterinary Parasitology, Babol Branch, Islamic Azad University, Babol, Iran*

**Keywords:** Pelargonium roseum, Avian trichomoniasis, Metronidazole, Trichomonas gallinae, Pigeon

## Abstract

**Objective:**

*Pelargonium roseum *Willd. (Geraniaceae) is widely grown as an ornamental plant due to its strong pleasant rose-like odor. The present study evaluates the antitrichomonal effect of *P. roseum *essential oil (EO) against *Trichomonas gallinae* both *in vitro* and *in vivo* and compares it to that of metronidazole (MTZ) as a standard antitrichomonal drug.

**Materials and Methods:**

*In vitro* assays were accomplished in multi-well plates containing MTZ and EO at final concentrations of 2.5, 5, 10, 20, 50, and 100 μg/mL. *In vivo* assay was carried out on 40 experimentally infected pigeons receiving MTZ and EO at doses of 25 and 50 mg/kg.

**Results:**

The 24-hr MIC of MTZ was 10 µg/mL, while for EO it was 20 µg/mL. Treatment with MTZ 50 mg/kg after 4 days led to full recovery of infected pigeons however EO 50 mg/kg resulted in the same outcome after 5 days. No mortality or clinical side effects were seen in treated birds.

**Conclusion:**

The present study introduced *P. roseum *EO as a potent natural antitrichomonal agent effective against *T. gallinae*. Bioactive components of *P. roseum* can be used as potential therapeutic compounds in development of novel antitrichomonal drugs.

## Introduction

*Pelargonium roseum*

Willd. (Geraniaceae), is indigenous to Southern Africa, but due to its strong pleasant rose-like odor, it is widely grown as an ornamental plant in almost all parts of the world. *P. roseum *has woody straight stems with short rough hairs covering leaves which give the plant a sweet scent (Lis-Balchin et al., 1996 ▶, Carmen and Hancu, 2014 ▶). Several bioactivities including antimicrobial, analgesic and anti-inflammatory effects were attributed to* P. roseum *( Rezai et al., 2008 ▶; Carmen and Hancu, 2014 ▶). The antimicrobial efficacy of *P. roseum *essential oil was tested on several Gram-negative and Gram-positive bacteria as well as fungi. It was demonstrated that the essential oil of *P. roseum *possesses antibacterial and antifungal activities (Carmen and Hancu, 2014 ▶). 

*Trichomonas gallinae*, which is considered the cause of avian trichomoniasis is a flagellate located in the upper digestive and occasionally in the respiratory tracts of a large variety of birds, mainly in the orders Columbiformes and Falconiformes (Boal et al., 1998 ▶; Rouffaer et al., 2014 ▶). Birds with avian trichomoniasis present caseous lesions within the anterior digestive tract. The lesions range from mild, often subclinical infections, to sever inflammation which can be acute and fatal, and lead to death by starvation due to the obstruction of the lumen of the esophagus (Gerhold et al., 2008 ▶). *T. gallinae* has significant health and economic impacts on the poultry industry especially pigeons and game birds rearing and breeding (Stockdale et al., 2015 ▶) and is considered a major contributing factor to the regulation and even decline of avian populations. The drugs of choice for the treatment of trichomoniasis are nitroimidazoles. Subtherapeutic doses and preventive use of these drugs against trichomoniasis, have resulted in emergence of resistant strains of* T. gallinae* (Lumeij and Zwijnenberg, 1990 ▶).

Bearing all this in mind, the present study was designed to evaluate antitrichomonal effect of *P. roseum *essential oil against *T. gallinae* both *in vitro* and *in vivo* and to compare its effects to those of metronidazole as a standard antitrichomonal drug.

## Materials and Methods

**Materials**

For preparation of *P. roseum* essential oil (EO), aerial parts of the plant were collected from a herb garden (Kashan, Iran) during 2014. The identity of the plant was confirmed by professor Rahimian, Sari Agricultural and Natural Resources University (Sari, Iran). By using a Clevenger type apparatus, the essential oil of the plant was obtained by hydrodistillation. Gas chromatographic mass spectrometric (GC-MS) analysis was performed using a Shimadzu GC-9A with helium as the carrier gas on a DB-5 column (30 m × 0.25 mm i.d; film thickness 0.25 mm). GC–MS analysis was carried out on a Varian 3400 GC–MS system with oven temperature set at 40-250°C at a rate of 4°C; transfer line temperature, 260°C; carrier gas, helium with a linear velocity of 31.5 cm/s; split ratio, 1/60; ionization energy, 70 eV; scan time, 1 s: mass range, 40-300 amu.

Metronidazole (MTZ), (Alborz daru, Tehran, Iran) was used as a standard antitrichomonal agent. All other chemicals used in the present study were of analytical grade.

**Parasites**

*T. gallinae* were recovered by wet mount method from infected native pigeons. Forty native pigeons (6 to 8 weeks old) were purchased from local breeders in Babol city (Mazandaran Province, Iran). By using swabs moistened with saline solution, samples were taken from membranous lesions of oropharyngeal region of suspicious birds. To produce wet smears, swabs were rubbed over the surface of a microscope slide. Smears were then examined under a light microscope at X100 and X400 magnification to confirm *T. gallinae* as the causative agent of lesions. Parasite culture was prepared by immersing oral swabs in tryptone/ yeast extract/ maltose (TYM) medium supplemented with 10% fetal calf serum (Sigma, Germany) and incubated at 37°C (Sansano-Maestre et al., 2009 ▶). Cultures were observed over five consecutive days to check the growth of *T. gallinae*. Every 48 hr as the parasites in the logarithmic phase of growth presented more than 95% mobility and normal morphology, isolates were sub-cultured (Seddiek et al., 2014 ▶).

***In vitro*** **assay**

For the *in vitro* assay, we used the method described by Munoz et al., 1998 after making slight modifications. To examine the susceptibilities of *T. gallinae *toward *P. roseum *EO and MTZ, sterile multi-well plates were used to incubate the trophozoites with different concentrations of the essential oil and drug dilutions. A volume of 100 µL of culture medium containing 1× 10^4^ parasites was pipetted into each well. Also, prediluted MTZ and *P. roseum *EO were added to wells to give final concentrations of 2.5, 5, 10, 20, 50, and 100 μg/mL. Tween 20 (0.01% of final concentration) was used as a solubilizing agent for *in vitro* analysis. Control wells received only Tween 20 without any treatments. Subsequently, to generate anaerobic conditions, a layer of 50 µL of vaseline was added to wells based on the method developed by Munoz et al., 1998. All assays were done three times. The wells were examined using an inverted microscope every 24 hr for 3 successive days. The MIC was the lowest concentration of the drug in the well at which no motile parasite was observed. 

***In vivo*** **assay**

The protocol used for the *in vivo* study was in accordance with laboratory animal welfare guide of Pasteur Institute of Iran and has been approved by the ethics committee. Forty native pigeons ( >6 weeks old) were examined and confirmed to be free of *T. gallinae* then experimentally infected with Trichomonas by inoculation of 4 × 10^4^ trophozoites prepared in 1 mL of 48-hr culture medium. Seven days post-infection, birds were examined and those that were diagnosed as infected with *T. gallinae*, were randomly allocated into 5 groups as follows: the first group (CON) were infected but not medicated, EO 25 and EO 50 were the groups infected with *T. gallinae* and medicated with 25 and 50 mg/kg of *P. roseum* essential oil, respectively. MTZ 25 and MTZ 50 groups were infected with *T. gallinae* and medicated with 25 and 50 mg/kg of metronidazole, respectively. All of the treatments were administered orally (PO) for 7 successive days. Birds of different groups were located in separate wire cages and fed with semisolid mixed grains diet in order to prevent starvation due to difficulty in swallowing because of trichomoniasis. The numbers of motile trophozoites recovered from the infected birds, were determined every day for seven consecutive days. 

**Statistical analysis**

Analysis of variance (ANOVA) was used, followed by Newman Keul’s test as the *post hoc* test to evaluate differences among means (SPSS ver. 18). A p value less than 0.05 was considered statistically significant. The growth inhibition percentage was determined using the following equation: 

growth inhibition % = (A − B)/A × 100

where A is the mean number of trophozoites in the control group and B is the mean number of trophozoites in the test group (Seddiek et al., 2014 ▶).

## Results

***In vitro*** **results**

The results of *in vitro* study of anti-trichomonal activity of *P. roseum* essential oil and comparison of this activity with that of metronidazole as a standard anti-protozoal drug are shown in Table. 1. The results revealed high efficacy of *P. roseum* EO against *T. gallinae*. At the dose of 10 µg/mL, MTZ after 24-hr incubation resulted in no viable trophozoite in culture medium. The 24-hr MIC of *P. roseum* EO was 20 µg/mL. The 48 and 72-hr MIC values of MTZ were 5 and 2.5 µg/mL, respectively but these values for *P. roseum* were 10 and 5 µg/mL, respectively.

**Figure 1:**
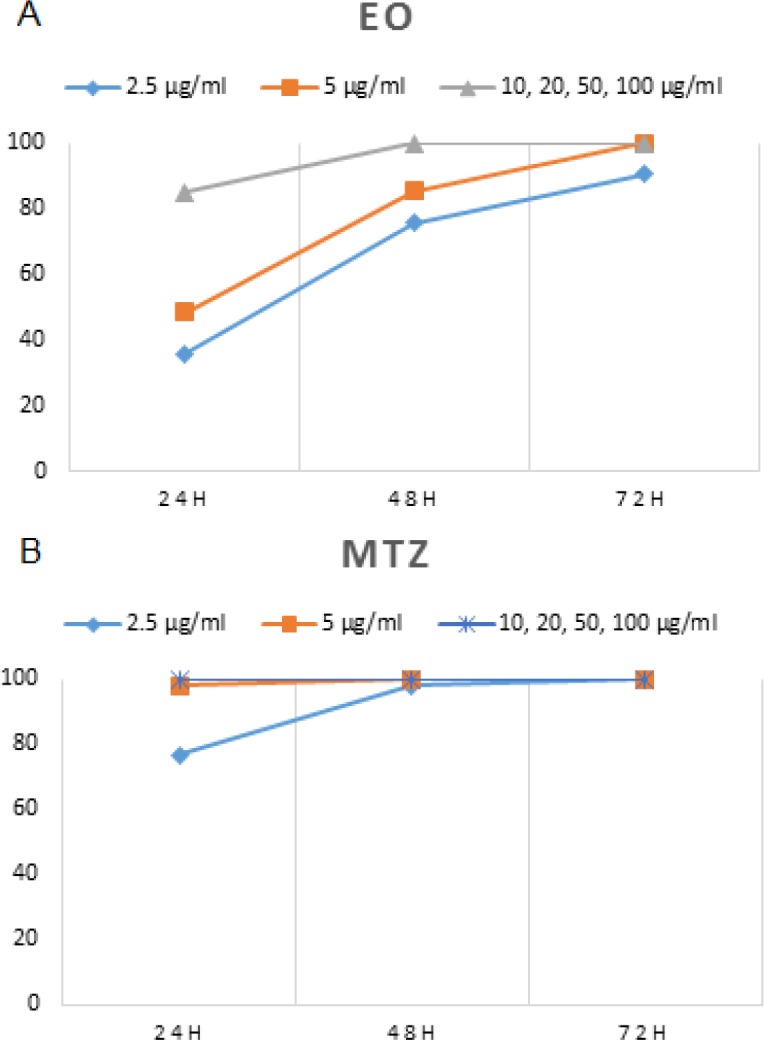
*In vitro* growth inhibition percentage (GI %) in *P. roseum *EO (A) and MTZ (B)*-*treated groups at 24-hr intervals.

Results of growth inhibition percentage (GI %) in MTZ and *P. roseum-*treated groups at 24-hr intervals are shown in Figure. 1. It was shown that there was significant difference in GI% between MTZ and *P. roseum *EO-treated groups and the control group. Based on dose- GI% graphs, 2.5, 5, and 10 µg/mL of MTZ and *P. roseum *EO resulted in different responses of GI% (Figure. 1). 

**Table 1:** *In vitro* anti-trichomonal activity of metronidazole (MTZ) and *P. roseum* essential oil (EO) against trophozoites of *T. gallinae*.

Time (Hr)	Groups												
	CON	MTZ (µg/ml)						*P. roseum *EO (µg/ml)					
		2.5	5	10	20	50	100	2.5	5	10	20	50	100
24	7.93 ± 0.04	1.84± 0.13^ab^	0.15 ± 0.04^a^	0 ^a^	0 ^a^	0 ^a^	0^a^	5.1 ± 0.28^ac^	4.08 ± 0.36 ^ac^	1.18 ± 0.05^ab^	0 ^a^	0 ^a^	0 ^a^
48	9.41 ± 0.28	0.19 ± 0.03 ^a^	0^a^	0 ^a^	0 ^a^	0 ^a^	0 ^a^	2.29 ± 0.41^ab^	1.38 ± 0.2^ab^	0 ^a^	0 ^a^	0^a^	0 ^a^
72	9.04 ± 0.32	0 ^a^	0^a^	0 ^a^	0 ^a^	0 ^a^	0 ^a^	0.85 ± 0.13^ab^	0 ^a^	0 ^a^	0 ^a^	0 ^a^	0 ^a^

Data are presented as mean ± SEM. Mean number of trophozoites×10^4^.

a, b, c different letters in the same row indicate statistical significant difference (p<0.05).

**Table 2:** *In vivo* anti-trichomonal activity of metronidazole (MTZ) and *P. roseum* essential oil (EO) against *T. gallinae*.

Days	Groups				
	CON	MTZ (25 mg/kg)	MTZ (50 mg/kg)	*P. roseum *EO (25 mg/kg)	*P. roseum *EO (50 mg/kg)
1	130.56 ± 4.67	137.2 ± 3.76	140 ± 2.33	130.8 ± 3.43	140.16 ± 4.36
2	140.64 ± 5.55	128.56 ± 3.17	66.42 ±3.28^a^	107.4 ± 4.04	83.83 ± 1.37 ^a^
3	135.44± 4.64	84.08 ±1.50 ^a^	8.02 ± 0.02^ab^	83.32 ± 1.06 ^a^	32.42 ± 0.35^ac^
4	132.2 ± 5.9	36.73 ± 5.04 ^a^	0.42 ± 0.01^ab^	64.01 ± 0.5 ^a^	10.05 ± 0.12^ac^
5	136.16 ± 2.75	26.8 ± 1.32 ^a^	0^ab^	42.2± 0.04 ^a^	0.32± 0.11^ab^
6	126.56 ± 0.68	13.2 ± 0.66 ^a^	0^ab^	20.12 ± 3.35 ^a^	0^ab^
7	127.28 ± 2.21	6.41 ± 0.26 ^a^	0^a^	13.83 ± 1.37 ^a^	0 ^a^
8	115.68 ± 2.32	3.2 ± 0.41 ^a^	0^a^	2.57 ± 1.18 ^a^	0 ^a^

Data are presented as Mean ± SEM. Mean number of trophozoites×10^4^.

a, b, c different letters in the same row indicate statistical significant difference (p<0.05).

***In vivo*** **results**

*In vivo* assay demonstrated effectiveness of MTZ and *P. roseum *EO against *T. gallinae* in experimentally infected pigeons (Table 2). Seven days post-inoculation of *T. gallinae* (day 1), before initiation of treatments, number of trophozoites recovered from birds were measured and no significant difference was seen among different groups. On the second day of the experiment, treatment with MTZ and EO at the dose of 50 mg/kg resulted in significant reduction of trophozoites in comparison to control group (p<0.05). MTZ at the dose of 50 mg/kg on the 3^rd^ and 4^th^ day of the treatment resulted in significant reduction in the number of *T. gallinae* in comparison to all other groups (p<0.05). On the 5^th ^day, no motile trophozoite was recovered from birds treated with MTZ 50 mg/kg treated. One day later (the 6^th^ day), treatment with EO 50 mg/kg led to full recovery of infected pigeons. It should be noted that MTZ and EO at the dose of 25 mg/kg even after 1 week of treatment did not cause full recovery. No mortality was recorded for treatment groups and no clinical side effects were observed in treated birds.

**Figure 2:**
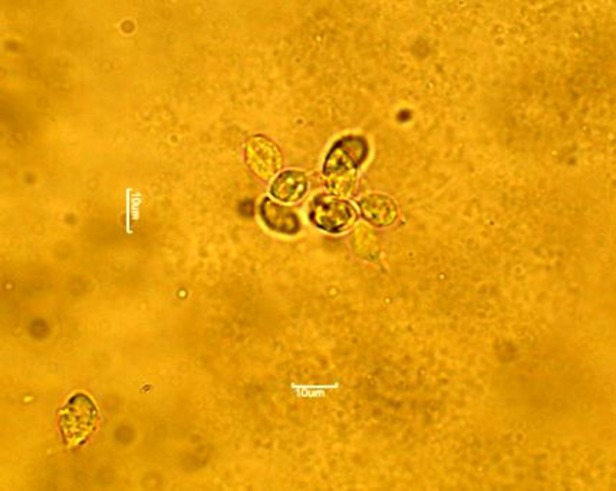
*T* *.* *gallinae* recovered from infected pigeons.

**Chemical composition of** ***P. roseum*** **EO**

Major constituents of *P. roseum* EO were β- citronellol (29%), geraniol (18.5%), and linalool (5.72%), as shown in Table 3.

**Table 3:** Chemical composition of *P. roseum *essential oil.

**Compounds**	**Retention Index**	**% Constituents of ** *** P. roseum *** **EO**
**a-pinene**	934	0.7
**β-pinene**	973	0.1
**β-Myrcene**	990	0.15
**a-Phellandrene**	1004	0.24
**p-Cymene**	1019	0.1
**D-Limonene**	1025	0.68
**β-Ocimene**	1033	0.2
**γ-Terpinene**	1053	0.1
**Linalool oxide**	1065	0.6
**Linalool**	1093	5.72
**Cis-Rose oxide**	1107	1.44
**Trans-Rose oxide**	1121	0.55
**β-Terpineol**	1139	0.27
**Menthone**	1150	2.55
**iso-Menthone**	1159	4.22
**Menthol**	1166	0.18
**Terpinene 4-oL**	1172	0.5
**α-Terpineol**	1185	1.3
**β-Citronellol**	1225	28.96
**Geraniol**	1251	18.53
**α-Cubebene**	1346	1.25
**Citronellyl acetate**	1354	3.51
**β-Bourbonene**	1388	2.14
**β-elemene**	1389	0.1
**α-Gurjunene**	1409	0.35
**β-Caryophyllene**	1420	2.6

## Discussion

Preventive treatment with nitroimidazoles have resulted in the emergence of resistant isolates of *T. gallinae* which are potentially serious threats endangering birds’ lives particularly wild birds. Nitroimidazole-resistant isolates of *T. gallinae* have been reported from different parts of the world including Belgium, Spain, the United States and Iran (Munoz et al., 1998 ▶; Gerhold et al., 2008 ▶; Rouffaer et al., 2014 ▶; Tabari et al, 2017a ▶). In spite of the fact that nitroimidazole-resistant strains of *T. gallinae* are becoming prevalent, very few research has focused on finding alternative antitrichomonal agents effective against *T. gallinae*. Youssefi et al. (2017) ▶ evaluated the effects of essential oil of *Artemisia sieberi *against *T. gallinae*. Treatment with *A. sieberi* 10 μg/ml and metronidazole 20 μg/ml resulted in no viable trophozoite in culture medium after 24 hr (Youssefi et al., 2017 ▶). Also, Tabari et al. (2017a) demonstrated the antitrichomonal effects of *Peganum harmala* alkaloid extract against *T. gallinae*, both *in vitro* and *in vivo*, and compared the effects of of metronidazole, as a conventional antitrichomonal medication with those of harmine and harmaline, the two alkaloids present in *P. harmala*. It was shown that *P. harmala* alkaloid extract can be considered a potent natural anti-trichomonal agent and an efficacious alternative for treating metronidazole-resistant isolates of *T. gallinae* (Tabari et al., 2017a ▶). Seddik and coworkers (2014) reported that garlic was as effective as MTZ in inhibiting *T. gallinae* trophozoites’ growth both *in vitro* and *in vivo*. They also observed side effects like cytotoxicity, carcinogenic effects and neurological dysfunction following MTZ administration, and recommended garlic as a safe alternative for prophylactic and therapeutic uses in case of trichomoniasis (Seddiek et al., 2014 ▶). 

They found that 4-day treatment with garlic 200 mg/kg was effective for treatment of infected pigeons (Seddiek et al., 2014 ▶). In the present study, *P. roseum* 5-day treatment with EO at a dose much lower than 50 mg/kg, resulted in full recovery of infected birds. Seddik et al. (2014) reported the 24-hr MIC of garlic extract 75 µg/mL while this value for *P. roseum* was 20 µg/mL. Comparison of these results reveal a probably higher efficacy of *P. roseum* against *T. gallinae*. Fakhrie-Kashan showed that the alcoholic and aqueous extracts of *P. roseum* had inhibitory effects on the growth of *Trichomonas* *vaginalis* trophozoeites (Fakhrie-Kashan et al., 2014). The IC_50_ value of the aqueous and alcoholic extracts of *P. roseum* and MTZ after 24 hr, were 54.67, 27.63 and 0.0326 µg/mL, respectively. 

The gas chromatographic (GC) analysis of *P. roseum* used in the present study revealed that β- citronellol (29%) and geraniol (18.5%) were the major constituents of the essential oil. Toxicity of β- citronellol and geraniol in *Culex pipiens* complex have been evaluated and ovicidal, larvicidal and moderate knock-down effect of these compounds in adults has been reported (Tabari et al., 2017). Antitrichomonal activity of *P. roseum *EO against *T. gallinae *is probably due to its citroneloll, geraniol and linalool contents; however, further studies for elucidation of the most effective components and their mechanism of action are still required. Data obtained in this study introduced *P. roseum* as a potent natural antitrichomonal agent effective against *T. gallinae*. Bioactive components of *P. roseum* can be used as leading compounds in development of novel antitrichomonal drugs. In addition, further studies on probable inhibitory effects of *P. roseum* against *T. vaginalis* are also recommended. 
